# The association between subjective impact and the willingness to adopt healthy dietary habits after experiencing the outbreak of the 2019 novel coronavirus disease (COVID-19): a cross-sectional study in China

**DOI:** 10.18632/aging.103929

**Published:** 2020-11-05

**Authors:** Ying Xu, Zhixue Li, Weijun Yu, Xiangyang He, Yan Ma, Fengmin Cai, Zheng Liu, Rencheng Zhao, Dewang Wang, Yan-Fang Guo, Jialong Chen

**Affiliations:** 1 Bao'an District Hospital for Chronic Diseases Prevention and Cure, Shenzhen 518100, China; 2 School of Public Health, Guangdong Medical University, Dongguan 523808, China

**Keywords:** COVID-19, epidemic, diet, subjective impact, cross-sectional study

## Abstract

To investigate the associations between subjective perception of impacts and willingness to change dietary habits in China after experiencing the outbreak of the 2019 novel coronavirus disease (COVID-19), an online questionnaire survey was carried out and 22,459 respondents in mainland China participated in the study, with an average age of 27.9±7.8 years old. Of them, 84.5% self-reported epidemic concern (middle or above), and 60.2%, 66.3% and 66.8% self-reported impact (middle or above) on psychology, life, work respectively. 31.9%, 46.0% and 41.0% of respondents reported their willingness to reduce their dietary intakes of salt, fried foods, and sugary foods, respectively. The stratified analysis of multinomial logistic regression models showed that, respondents with higher psychological impact were more likely to increase their dietary intake of salt, fried foods, sugary foods. Except as aforesaid, most respondents with higher epidemic concerns and higher impacts on psychology, life, work were more likely to reduce eating salt, fried foods, sugary foods. After the epidemic, early stage of positive improvement to a proper diet was observed, whereas the opposite tendency was also found in some respondents with higher impact on psychology. Thus, there is an urgent need for health care and lifestyle intervention policies for different subgroups.

## INTRODUCTION

Since the outbreak of the 2019 novel coronavirus (COVID-19), the disease has swept across globally, causing more than 8,061,550 confirmed cases and 440,290 fatalities (by 10:00 CEST, 17 June 2020) [1]. The outbreak of the disease led to psychological impact like fears, sadness, anxiety, and depression of the people on how to manage the disease during the hard time [2]. Yeen Huang [3] et al. reported that the prevalence rates of generalized anxiety disorder, depression and sleep disorder in the ordinary residents during the epidemic period were 35.1%, 20.1% and 18.2%, respectively. Besides, it is imposing social and socio-economic impacts in China, including disproportionate time of lockdown, prevalent disruption of the global chain supply due to the closing their boarder, slowdown of the investment, loss of revenue due to debt, increment in health spending cost, shortage of food and drugs, decrement of business travel and tightening domestic financial markets [4, 5]. Moreover, psychosocial tolls were associated with unhealthy lifestyle behaviors, including physical and social inactivity, poor sleep quality, unhealthy diet behaviors, and unemployment [6].

Patients with chronic diseases are more likely to be infected with COVID-2019 and have a higher mortality rate than healthy people [7]. Epidemiological data of 72,314 cases from the Chinese Centre for Disease Control and Prevention indicated that most of the COVID-2019 victims suffered from basic diseases, such as hypertension, cardiovascular and cerebrovascular diseases, and diabetes [8]. A nationwide analysis [9] of comorbidity and its impact on COVID-19 in China showed that patients with chronic obstructive pulmonary disease (Hazard Ratio, HR 2.681, 95% CI 1.42–5.05), diabetes (HR 1.59, 95% CI 1.03–2.45), hypertension (HR 1.58, 95% CI 1.07–2.32), and malignancy (HR 3.50, 95% CI 1.60–7.64) were more likely to reach to the composite endpoints than those without them. Compared with patients without comorbidity, the HR (95% CI) was 1.79 (95% CI 1.16–2.77) among patients with at least one comorbidity and 2.59 (95% CI 1.61–4.17) among patients with two or more comorbidities [9].

In order to fight COVID-19, some effective personal hygiene practices were adopted by the general public. Lorene M. Nelson et al. reported that most (95.7%) respondents were making changes to their lifestyle, mainly including more hand washing (93.1%), avoiding social gatherings (89.0%), and stockpiling food and supplies in response to COVID-19 (74.7%) [10]. Kin On Kwok et al. also reported that most respondents (>89%) adopted enhanced personal hygiene practices (including wearing masks, cleaning their hands, and adopting better coughing and sneezing etiquette) and avoided traveling [11]. Obviously, people around the world have tended to change their lifestyles in response to the virus. However, food consumption and meal patterns (the type of food, eating out of control, snacks between meals, number of meals) were unhealthier during confinement due to COVID-19 pandemic [12]. Whether health-related dietary habits of food groups (e.g., dietary intakes of salt, fried foods, and sugary foods), which has been considered effective to decrease the risk of chronic diseases [13] to reduce serious outcomes of COVID-19 infections, were or will be adopted by general residents is still unknown.

Therefore, this study tried to explore whether the outbreak of COVID-19, especially subjective perception of the epidemic concern and impacts on the situation of psychology, life, work, or study, which changed the health-related diet habits of Chinese people. The results of this study provide a basis for our targeted health education in the future.

## RESULTS

### General information

From 0:00 on April 8, 2020, Wuhan officially lifted the control measures. After 76 days of "war epidemic", Wuhan officially restarted. To investigate the association between subjective impact and the willingness to adopt healthy dietary habits after experiencing the outbreak of the 2019 novel coronavirus disease, the online questionnaire started on April 25.

A total of 22,459 subjects were included in this study, including 14,204 males (63.2%) and 8,255 females (36.8%). Among them, 11,182 (49.8%) were married, and 10,567 (47.1%) were unmarried; 20,650 (91.9%) were in senior high school and above and 1,809 (8.1%) in junior high school and below; only 711 (3.2%) were medical workers. The mean age and BMI were 27.9±7.8 (years) and 22.1±4.9, respectively. During the outbreak of COVID-19, 14,069 (62.6%) lived in Guangdong Province, 292 (1.3%) lived in Hubei, the area with severe epidemics in China, and 8,098 (36.1%) in other provinces (Tables 1 and 2).

**Table 1:** Demographic characteristics of the participants.

**Characteristic or indicator**	**Male (N=14,204)**	**Female (N=8,255)**	**Total (N=22,459)**	**t or χ^2^**	***P* value**
**Characteristics**					
Age	26.8±7.8	29.7±7.5	27.9±7.8	-27.638	<0.001
BMI	22.4±4.9	21.4±4.8	22.1±4.9	14.422	<0.001
Main living place					
Guangdong Province	5,298 (64.2%)	8,771 (61.8%)	14,069 (62.6%)	14.982	0.001
Hubei Province	92 (1.1%)	200 (1.4%)	292 (1.3%)		
Other	2,865 (34.7%)	5,233 (36.8%)	8,098 (36.1%)		
Occupation					
Medical workers	286 (3.5%)	425 (3.0%)	711 (3.2%)	3.801	0.051
Other	7,969 (96.5%)	13,779 (97.0%)	21,748 (96.8%)		
Education level					
Primary schools and below	98 (1.2%)	238 (1.7%)	336 (1.5%)	60.154	<0.001
Junior high school	554 (6.7%)	919 (6.5%)	1,473 (6.6%)		
High school or technical secondary school	1,956 (23.7%)	3,918 (27.6%)	5,874 (26.2%)		
College	2,567 (31.1%)	3,940 (27.7%)	6,507 (29.0%)		
Bachelor degree or above	3,080 (37.3%)	5,189 (36.5%)	8,269 (36.8%)		
Marital status					
Married	5,161 (62.5%)	6,021 (42.4%)	11,182 (49.8%)	847.383	<0.001
Unmarried	2,886 (35.0%)	7,681 (54.1%)	10,567 (47.1%)		
Other (including cohabitation, divorced, widowed, separation)	208 (2.5%)	502 (3.5%)	710 (3.2%)		

**Table 2:** Investigated questions on subjective impact, baseline of dietary habit, and willingness to change dietary intake.

Investigated questions
**Subjective impact**
Epidemic concern: “How concerned do you feel about COVID-19?”
Impact on psychology: “What about the affection of your psychological status by COVID-19 ?”
Impact on life: “What about the affection of your daily life by COVID-19 ?”
Impact on work: “What about the affection of your work or study by COVID-19 ?”
**Baseline of dietary habit**
Salt intake: “Before COVID-19, What about your dietary intake of salt?”
Intake frequency of fried food: “Before COVID-19, how often did you normally eat fried foods?”
Intake frequency of sugary foods: “Before COVID-19, how often did you normally eat sugar or sugary foods (including sugary drinks, desserts, biscuits, sweets, fruit products, dairy desserts etc)?”
**Willingness to change dietary intake**
Salt: “Do you plan or are you changing salt intake after the COVID-19 epidemic”
Fried food: “Do you plan or are you changing the intake of fried food after the COVID-19 epidemic”
Sugary foods: Do you plan or are you changing the intake of sugary foods after the COVID-19 epidemic”

### Subjective impact

Nearly one-third and two-fifths of self-reported status of epidemic concern were high (32.5%) and higher (42.5%), respectively. Over 60.0% self-reported a subjective impact (middle or above) on psychology (60.2%), life (66.3%), and work (66.8%). In different genders, the difference in subjective impacts was statistically significant (P<0.05) (Table 3).

**Table 3:** Subjective impact, baseline of dietary habit, and willingness to change dietary intake after the outbreak of COVID-19.

**Characteristic or indicator**	**Male (N=14,204)**	**Female (N=8,255)**	**Total (N=22,459)**	**t or χ^2^**	***P* value**
**Subjective impact**					
Epidemic concern					
None	91 (1.1%)	320 (2.3%)	411 (1.8%)	162.593	<0.001
Low	953 (11.5%)	2,108 (14.8%)	3,061 (13.6%)		
Medium	634 (7.7%)	1,496 (10.5%)	2,130 (9.5%)		
High	2,815 (34.1%)	4,493 (31.6%)	7,308 (32.5%)		
Higher	3,762 (45.6%)	5,787 (40.7%)	9,549 (42.5%)		
Impact on psychology					
None	677 (8.2%)	1,175 (8.3%)	1,852 (8.2%)	6.039	0.196
Low	2,646 (32.1%)	4,447 (31.3%)	7,093 (31.6%)		
Medium	2,677 (32.4%)	4,792 (33.7%)	7,469 (33.3%)		
High	1,605 (19.4%)	2,639 (18.6%)	4,244 (18.9%)		
Higher	650 (7.9%)	1,151 (8.1%)	1,801 (8.0%)		
Impact on life					
None	562 (6.8%)	1,007 (7.1%)	1,569 (7.0%)	6.899	0.141
Low	2,229 (27.0%)	3,766 (26.5%)	5,995 (26.7%)		
Medium	2,841 (34.4%)	4,939 (34.8%)	7,780 (34.6%)		
High	1,904 (23.1%)	3,142 (22.1%)	5,046 (22.5%)		
Higher	719 (8.7%)	1,350 (9.5%)	2,069 (9.2%)		
Impact on work					
None	598 (7.2%)	1,104 (7.8%)	1,702 (7.6%)	21.643	<0.001
Low	2,126 (25.8%)	3,641 (25.6%)	5,767 (25.7%)		
Medium	2,782 (33.7%)	4,755 (33.5%)	7,537 (33.6%)		
High	1,912 (23.2%)	3,036 (21.4%)	4,948 (22.0%)		
Higher	837 (10.1%)	1,668 (11.7%)	2,505 (11.2%)		
**Baseline of dietary habit**					
Salt intake				
High	1,537 (18.6%)	3,317 (23.4%)	4,854 (21.6%)	206.927	<0.001
Medium	5,076 (61.5%)	9,013 (63.5%)	14,089 (62.7%)		
Low	1,642 (19.9%)	1,874 (13.2%)	3,516 (15.7%)		
Intake frequency of fried food				
Every day	900 (10.9%)	2,091 (14.7%)	2,991 (13.3%)	200.670	<0.001
4–6 days/week	2,068 (25.1%)	4,106 (28.9%)	6,174 (27.5%)		
2–3 days/week	2,615 (31.7%)	4,515 (31.8%)	7,130 (31.7%)		
<1 days/week	2,672 (32.4%)	3,492 (24.6%)	6,164 (27.4%)		
Intake frequency of sugary foods				
Every day	1,567 (19.0%)	3,108 (21.9%)	4,675 (20.8%)	94.877	<0.001
4–6 days/week	2,305 (27.9%)	4,414 (31.1%)	6,719 (29.9%)		
2–3 days/week	2,980 (36.1%)	4,789 (33.7%)	7,769 (34.6%)		
<1 days/week	1,403 (17.0%)	1,893 (13.3%)	3,296 (14.7%)		
**Willingness to change dietary intake**				
Salt				
Unchange	3,832 (46.4%)	6,592 (46.4%)	10,424 (46.4%)	281.684	<0.001
Increase	978 (11.8%)	2,558 (18.0%)	3,536 (15.7%)		
Decrease	3,066 (37.1%)	4,091 (28.8%)	7,157 (31.9%)		
Uncertain	379 (4.6%)	963 (6.8%)	1,342 (6.0%)		
Fried food					
Unchange	2,251 (27.3%)	4,346 (30.6%)	6,597 (29.4%)	280.012	<0.001
Increase	1,297 (15.7%)	3,097 (21.8%)	4,394 (19.6%)		
Decrease	4,371 (52.9%)	5,963 (42.0%)	10,334 (46.0%)		
Uncertain	336 (4.1%)	798 (5.6%)	1,134 (5.0%)		
Sugary foods					
Unchange	2,547 (30.9%)	4,630 (32.6%)	7,177 (32.0%)	290.888	<0.001
Increase	1,358 (16.5%)	3,350 (23.6%)	4,708 (21.0%)		
Decrease	3,926 (47.6%)	5,274 (37.1%)	9,200 (40.0%)		
Uncertain	424 (5.1%)	950 (6.7%)	1,374 (6.1%)		

### Baseline of dietary habits, and willingness to change dietary habits

Fewer respondents reported that they were used to a high-salt diet (21.6%), whereas almost two-fifths and half reported that their intake frequency of fried food was 4–6 days/week or above (40.8%) and that their intake frequency of sugary foods was 4–6 days/week or above (50.7%). The difference in dietary habits between different genders was statistically significant (P<0.05) (Table 3).

Nearly 30% reported their willingness to reduce their salt intake (31.9%) and more than 40% reported their willingness to reduce their intake of fried foods (46.0%), sugary foods (41.0%). Significant differences of willingness to change dietary habits were observed in both different genders and different baselines of dietary habits (P<0.05) (Tables 3, 4).

**Table 4:** The analysis of willingness to change dietary habits under the status of baseline.

**Dietary custom**	**Unchange (N, %)**	**Increase (N, %)**	**Decrease (N, %)**	**Uncertain (N, %)**	**Total (N, %)**	**χ^2^**	***P-value***
**Salt**							
High	1,872 (38.6)	737 (15.2)	2,035 (41.9)	210 (4.3)	4,854 (100)	482.027	<0.001
Medium	6,574 (46.7)	2,467 (17.5)	4,134 (29.3)	914 (6.5)	14,089 (100)		
Low	1,978 (56.3)	332 (9.4)	988 (28.1)	218 (6.2)	3,516 (100)		
Total	10,424 (46.4)	3,536 (15.7)	7,157 (31.9)	1,342 (6.0)	22,459 (100)		
**Fried foods**							
Every day	1,902 (63.6)	467 (15.6)	557 (18.6)	65 (2.2)	2,991 (100)	4,700.632	<0.001
4–6 days/week	1,126 (18.2)	2,270 (36.8)	2,597 (42.1)	181 (2.9)	6,174 (100)		
2–3 days/week	1,207 (16.9)	1,375 (19.3)	4,196 (58.8)	352 (4.9)	7,130 (100)		
<1 days/week	2,362 (38.3)	282 (4.6)	2,984 (48.4)	536 (8.7)	6,164 (100)		
Total	6,597 (29.4)	4,394 (19.6)	10,334 (46.0)	1,134 (5.0)	22,459 (100)		
**Sugary foods**							
Every day	2,795 (59.8)	742 (15.9)	998 (21.3)	140 (3)	4,675 (100)	3,995.560	<0.001
4–6 days/week	1,329 (19.8)	2,421 (36.0)	2,706 (40.3)	263 (3.9)	6,719 (100)		
2–3 days/week	1,843 (23.7)	1,320 (17.0)	4,108 (52.9)	498 (6.4)	7,769 (100)		
<1 days/week	1,210 (36.7)	225 (6.8)	1,388 (42.1)	473 (14.4)	3,296 (100)		
Total	7,177 (32.0)	4,708 (21.0)	9,200 (41.0)	1,374 (6.1)	22,459 (100)		

### Univariate analysis of associations between subjective impact and willingness to change dietary habits

Those who self-reported higher epidemic concerns and higher impacts on psychology, life, work were more likely to reduce their intakes of salt, fried foods, and sugary foods (P<0.05). However, psychological impact was positively associated with willingness to increase fried foods, and sugary foods (P<0.05) (Table 5).

**Table 5:** Univariate analysis of associations between subjective impact and willingness to change dietary habits.

**Subjective impact**	**Salt intake**					**Fried foods intake**				**Sugary foods intake**			
	**Unchange (N, %)**	**Increase (N, %)**	**Decrease (N, %)**	**Uncertain (N, %)**		**Unchange (N, %)**	**Increase(N, %)**	**Decrease (N, %)**	**Uncertain (N, %)**		**Unchange (N, %)**	**Increase (N, %)**	**Decrease (N, %)**	**Uncertain (N, %)**
**Epidemic concern**														
None	149 (36.3)	151 (36.7)	92 (22.4)	19 (4.6)		132 (32.1)	139 (33.8)	102 (24.8)	38 (9.2)		118 (28.7)	134 (32.6)	112 (27.3)	47 (11.4)
Low	1,134 (37.0)	1,009 (33.0)	769 (25.1)	149 (4.9)		729 (23.8)	987 (32.2)	1,172 (38.3)	173 (5.7)		796 (26.0)	1,024 (33.5)	1,061 (34.7)	180 (5.9)
Medium	872 (40.9)	599 (28.1)	522 (24.5)	137 (6.4)		597 (28.0)	565 (26.5)	827 (38.8)	141 (6.6)		603 (28.3)	576 (27.0)	783 (36.8)	168 (7.9)
High	3,639 (49.8)	854 (11.7)	2,347 (32.1)	468 (6.4)		2,293 (31.4)	1,231 (16.8)	3,401 (46.5)	383 (5.2)		2,508 (34.3)	1,332 (18.2)	3,005 (41.1)	463 (6.3)
Higher	4,630 (48.5)	923 (9.7)	3427 (35.9)	569 (6.0)		2,846 (29.8)	1,472 (15.4)	4,832 (50.6)	399 (4.2)		3,152 (33.0)	1,642 (17.2)	4,239 (44.4)	516 (5.4)
χ^2^	1,457.555					693.494					566.776			
*P value*	<0.001					<0.001					<0.001			
**Impact on psychology**														
None	1,143 (61.7)	302 (16.3)	340 (18.4)	67 (3.6)		842 (45.5)	350 (18.9)	574 (31.0)	86 (4.6)		856 (46.2)	384 (20.7)	520 (28.1)	92 (5.0)
Low	3,624 (51.1)	1187 (16.7)	1,895 (26.7)	387 (5.5)		2,395 (33.8)	1,407 (19.8)	2,965 (41.8)	326 (4.6)		2,544 (35.9)	1,429 (20.1)	2,723 (38.4)	397 (5.6)
Medium	3,365 (45.1)	1148 (15.4)	2,452 (32.8)	504 (6.7)		1,968 (26.3)	1,442 (19.3)	3,633 (48.6)	426 (5.7)		2,246 (30.1)	1,538 (20.6)	3,167 (42.4)	518 (6.9)
High	1,582 (37.3)	646 (15.2)	1,763 (41.5)	253 (6.0)		946 (22.3)	825 (19.4)	2,260 (53.3)	213 (5.0)		1,043 (24.6)	950 (22.4)	1,999 (47.1)	252 (5.9)
Higher	710 (39.4)	253 (14.0)	707 (39.3)	131 (7.3)		446 (24.8)	370 (20.5)	902 (50.1)	83 (4.6)		488 (27.1)	407 (22.6)	791 (43.9)	115 (6.4)
χ^2^	590.412					515.152					403.928			
*P value*	<0.001					<0.001					<0.001			
**Impact on life**														
None	948 (60.4)	294 (18.7)	287 (18.3)	40 (2.5)		753 (48.0)	333 (21.2)	412 (26.3)	71 (4.5)		755 (48.1)	343 (21.9)	398 (25.4)	73 (4.7)
Low	2,911 (48.6)	1,164 (19.4)	1,635 (27.3)	285 (4.8)		1,883 (31.4)	1,322 (22.1)	2517 (42.0)	273 (4.6)		2,039 (34.0)	1,350 (22.5)	2,307 (38.5)	299 (5.0)
Medium	3,504 (45.0)	1,209 (15.5)	2,559 (32.9)	508 (6.5)		2,143 (27.5)	1,524 (19.6)	3,686 (47.4)	427 (5.5)		2,396 (30.8)	1,596 (20.5)	3,281 (42.2)	507 (6.5)
High	2,177 (43.1)	643 (12.7)	1,902 (37.7)	324 (6.4)		1,254 (24.9)	854 (16.9)	2,682 (53.2)	256 (5.1)		1,399 (27.7)	986 (19.5)	2,323 (46.0)	338 (6.7)
Higher	884 (42.7)	226 (10.9)	774 (37.4)	185 (8.9)		564 (27.3)	361 (17.4)	1,037 (50.1)	107 (5.2)		588 (28.4)	433 (20.9)	891 (43.1)	157 (7.6)
χ^2^	501.643					512.272					358.026			
*P value*	<0.001					<0.001					<0.001			
**Impact on work**														
None	1,035 (60.8)	323 (19.0)	296 (17.4)	48 (2.8)		795 (46.7)	376 (22.1)	455 (26.7)	76 (4.5)		791 (46.5)	377 (22.2)	447 (26.3)	87 (5.1)
Low	2,697 (46.8)	1,221 (21.2)	1,610 (27.9)	239 (4.1)		1,761 (30.5)	1,357 (23.5)	2,408 (41.8)	241 (4.2)		1,885 (32.7)	1,379 (23.9)	2,250 (39.0)	253 (4.4)
Medium	3,335 (44.2)	1,204 (16.0)	2,501 (33.2)	497 (6.6)		2,100 (27.9)	1,489 (19.8)	3,533 (46.9)	415 (5.5)		2,327 (30.9)	1,568 (20.8)	3,123 (41.4)	519 (6.9)
High	2,196 (44.4)	562 (11.4)	1,853 (37.4)	337 (6.8)		1,239 (25.0)	777 (15.7)	2,675 (54.1)	257 (5.2)		1,393 (28.2)	930 (18.8)	2,317 (46.8)	308 (6.2)
Higher	1,161 (46.3)	226 (9.0)	897 (35.8)	221 (8.8)		702 (28.0)	395 (15.8)	1,263 (50.4)	145 (5.8)		781 (31.2)	454 (18.1)	1,063 (42.4)	207 (8.3)
χ^2^	650.163					581.566					380.408			
*P value*	<0.001					<0.001					<0.001			

### Multivariable analysis of associations between subjective impact and willingness to change dietary habits

With a baseline of dietary habit as a stratification factor and unwillingness to change dietary habit as a reference group, and after adjustment for gender, age, BMI, education level, marital status, occupation, and main living place, the stratified analysis of the multinomial logistic regression models showed that respondents with higher psychological impacts were more likely to increase their dietary intakes of salt, fried foods, and sugary foods (P<0.05). For those with low-salt diets, higher epidemic concern (OR 0.75, 95% CI 0.60–0.95) and higher impact on work (OR 0.68, 95% CI 0.54–0.86) showed a negative correlation with the willingness to reduce salt intake. Our results shown that higher impact on life was positively correlated with willingness to reduce fried foods intake (OR 0.79, 95% CI 0.66–0.93) for those who ate less fried food (< 1 day / week). Furthermore, higher impact on life was positively correlated with willingness to increase sugary foods intake (OR 1.70, 95% CI 1.12–2.59) for those who ate less sugary foods (<1 day/week). Except as aforesaid, subjective impacts (including epidemic concern, impact on psychology, life, work) were positively correlated with willingness to reduce salt, fried foods and sugary foods in most respondents (P<0.05) (Tables 6–8).

**Table 6:** Multivariable analysis of associations between subjective impact and willingness to change salt intake.

**Intake frequency^a^**	**Willingness^b^**	**Subjective impact**	**OR (95% CI)**	***P-*value**
**High**	Increase	epidemic concern	0.31 (0.26, 0.38)	<0.001
		impact of psychology	1.68 (1.32, 2.14)	<0.001
		impact of life	1.00 (0.76, 1.31)	0.999
		impact of work or study	0.70 (0.54, 0.91)	0.008
	Decrease	epidemic concern	1.57 (1.31, 1.89)	<0.001
		impact of psychology	1.61 (1.34, 1.93)	<0.001
		impact of life	0.86 (0.70, 1.06)	0.150
		impact of work or study	1.48 (1.22, 1.79)	<0.001
	Uncertain	epidemic concern	1.30 (0.92, 1.84)	0.144
		impact of psychology	1.23 (0.86, 1.75)	0.262
		impact of life	0.85 (0.57, 1.26)	0.407
		impact of work or study	1.42 (0.99, 2.03)	0.058
**Medium**	Increase	epidemic concern	0.30 (0.27, 0.33)	<0.001
		impact of psychology	1.85 (1.61, 2.13)	<0.001
		impact of life	0.92 (0.79, 1.07)	0.271
		impact of work or study	0.66 (0.57, 0.76)	<0.001
	Decrease	epidemic concern	0.82 (0.75, 0.91)	<0.001
		impact of psychology	1.63 (1.46, 1.83)	<0.001
		impact of life	1.08 (0.95, 1.22)	0.232
		impact of work or study	1.00 (0.89, 1.12)	0.942
	Uncertain	epidemic concern	0.87 (0.74, 1.04)	0.127
		impact of psychology	1.18 (0.97, 1.43)	0.100
		impact of life	1.27 (1.04, 1.56)	0.019
		impact of work or study	1.11 (0.92, 1.34)	0.276
**Low**	Increase	epidemic concern	0.33 (0.25, 0.44)	<0.001
		impact of psychology	2.10 (1.51, 2.91)	<0.001
		impact of life	1.08 (0.75, 1.56)	0.679
		impact of work or study	0.84 (0.59, 1.18)	0.311
	Decrease	epidemic concern	0.75 (0.60, 0.95)	0.016
		impact of psychology	2.18 (1.74, 2.72)	<0.001
		impact of life	1.21 (0.94, 1.55)	0.138
		impact of work or study	0.68 (0.54, 0.86)	0.001
	Uncertain	epidemic concern	0.58 (0.39, 0.86)	0.006
		impact of psychology	1.71 (1.18, 2.49)	0.005
		impact of life	1.35 (0.89, 2.06)	0.159
		impact of work or study	1.29 (0.87, 1.92)	0.202

a Baseline of salt intake was regarded as a stratification factor

b Unwillingness to change salt intake was regarded as a reference group

c Subjective impacts were classified into dichotomous variables in the models (regarding “none,” “low,” and “medium” as 1 and others as 2), adjusting for other characteristic factors (gender, age, BMI, education level, marital status, occupation, and main living place)

**Table 7:** Multivariable analysis of associations between subjective impact and willingness to change fried foods.

**Intake frequency^a^**	**Willingness^b^**	**Subjective impact^c^**	**OR (95% CI)**	***P-*value**
**Everyday**	Increase	epidemic concern	0.82 (0.65, 1.04)	0.109
		impact of psychology	1.08 (0.79, 1.49)	0.631
		impact of life	1.07 (0.74, 1.55)	0.708
		impact of work or study	1.37 (0.97, 1.92)	0.074
	Decrease	epidemic concern	0.99 (0.78, 1.26)	0.933
		impact of psychology	1.09 (0.81, 1.46)	0.560
		impact of life	1.55 (1.10, 2.18)	0.013
		impact of work or study	1.73 (1.26, 2.37)	0.001
	Uncertain	epidemic concern	0.94 (0.53, 1.66)	0.829
		impact of psychology	1.25 (0.57, 2.75)	0.573
		impact of life	0.78 (0.31, 1.94)	0.586
		impact of work or study	1.15 (0.52, 2.56)	0.738
**4–6 days/week**	Increase	epidemic concern	0.56 (0.48, 0.67)	<0.001
		impact of psychology	1.55 (1.26, 1.91)	<0.001
		impact of life	1.01 (0.81, 1.26)	0.938
		impact of work or study	0.97 (0.78, 1.21)	0.813
	Decrease	epidemic concern	0.86 (0.73, 1.02)	0.085
		impact of psychology	1.37 (1.12, 1.68)	0.002
		impact of life	1.24 (1.00, 1.54)	0.051
		impact of work or study	1.34 (1.09, 1.65)	0.005
	Uncertain	epidemic concern	0.63 (0.45, 0.90)	0.010
		impact of psychology	1.28 (0.82, 1.99)	0.276
		impact of life	0.97 (0.60, 1.56)	0.893
		impact of work or study	1.09 (0.70, 1.72)	0.700
**2–3 days/week**	Increase	epidemic concern	0.50 (0.41, 0.60)	<0.001
		impact of psychology	1.66 (1.33, 2.07)	<0.001
		impact of life	1.00 (0.79, 1.26)	0.999
		impact of work or study	0.81 (0.65, 1.02)	0.073
	Decrease	epidemic concern	0.86 (0.73, 1.02)	0.083
		impact of psychology	1.39 (1.15, 1.67)	<0.001
		impact of life	1.04 (0.86, 1.26)	0.658
		impact of work or study	1.17 (0.98, 1.41)	0.080
	Uncertain	epidemic concern	0.54 (0.41, 0.71)	<0.001
		impact of psychology	1.25 (0.90, 1.74)	0.185
		impact of life	1.04 (0.74, 1.47)	0.814
		impact of work or study	1.18 (0.86, 1.64)	0.303
**<1 day/week**	Increase	epidemic concern	0.43 (0.32, 0.58)	<0.001
		impact of psychology	1.74 (1.23, 2.44)	0.002
		impact of life	0.92 (0.64, 1.33)	0.659
		impact of work or study	1.13 (0.80, 1.60)	0.491
	Decrease	epidemic concern	1.29 (1.09, 1.52)	0.003
		impact of psychology	1.44 (1.23, 1.68)	<0.001
		impact of life	0.79 (0.66, 0.93)	0.005
		impact of work or study	1.18 (1.01, 1.38)	0.038
	Uncertain	epidemic concern	0.60 (0.47, 0.76)	<0.001
		impact of psychology	1.64(1.26, 2.14)	<0.001
		impact of life	0.86 (0.65, 1.14)	0.301
		impact of work or study	1.10 (0.85, 1.43)	0.479

a Baseline of fried foods was regarded as a stratification factor

b Unwillingness to change fried foods intake was regarded a reference group

c Subjective impacts were classified into dichotomous variables in the models (regarding “none,” “low,” and “medium” as 1 and others as 2), adjusting for other characteristic factors (gender, age, BMI, education level, marital status, occupation, and main living place)

**Table 8:** Multivariable analysis of associations between subjective impact and willingness to change sugary foods.

**Intake frequency ^a^**	**Willingness^b^**	**Subjective impact^c^**	**OR (95% CI)**	**P-value**
**Everyday**	Increase	epidemic concern	0.67 (0.56, 0.82)	<0.001
		impact of psychology	1.46 (1.15, 1.86)	0.002
		impact of life	1.26 (0.96, 1.66)	0.098
		impact of work or study	0.97 (0.75, 1.25)	0.791
	Decrease	epidemic concern	1.06 (0.87, 1.28)	0.571
		impact of psychology	1.07 (0.86, 1.32)	0.556
		impact of life	1.27 (0.99, 1.62)	0.058
		impact of work or study	1.57 (1.26, 1.97)	<0.001
	Uncertain	epidemic concern	0.72 (0.48, 1.07)	0.104
		impact of psychology	0.84 (0.50, 1.40)	0.503
		impact of life	1.31 (0.76, 2.26)	0.337
		impact of work or study	1.30 (0.78, 2.16)	0.324
**4–6 days/week**	Increase	epidemic concern	0.57 (0.49, 0.67)	<0.001
		impact of psychology	1.84 (1.51, 2.24)	<0.001
		impact of life	1.06 (0.86, 1.30)	0.599
		impact of work or study	0.87 (0.72, 1.06)	0.179
	Decrease	epidemic concern	0.88 (0.75, 1.03)	0.115
		impact of psychology	1.61 (1.33, 1.95)	<0.001
		impact of life	1.12 (0.92, 1.37)	0.252
		impact of work or study	1.18 (0.98, 1.42)	0.087
	Uncertain	epidemic concern	0.73 (0.54, 1.01)	0.054
		impact of psychology	1.41 (0.98, 2.03)	0.068
		impact of life	1.40 (0.95, 2.05)	0.086
		impact of work or study	0.94 (0.65, 1.36)	0.736
**2–3 days/week**	Increase	epidemic concern	0.38 (0.32, 0.45)	<0.001
		impact of psychology	1.79 (1.46, 2.19)	<0.001
		impact of life	0.98 (0.79, 1.22)	0.865
		impact of work or study	0.86 (0.70, 1.06)	0.154
	Decrease	epidemic concern	0.74 (0.63, 0.86)	<0.001
		impact of psychology	1.52 (1.30, 1.79)	<0.001
		impact of life	0.98 (0.83, 1.16)	0.846
		impact of work or study	1.00 (0.86, 1.17)	0.988
	Uncertain	epidemic concern	0.60 (0.47, 0.77)	<0.001
		impact of psychology	1.07 (0.81, 1.42)	0.643
		impact of life	1.19 (0.89, 1.59)	0.236
		impact of work or study	1.11 (0.85, 1.46)	0.444
**<1 day/week**	Increase	epidemic concern	0.43 (0.30, 0.61)	<0.001
		impact of psychology	1.26 (0.85, 1.87)	0.243
		impact of life	1.70 (1.12, 2.59)	0.014
		impact of work or study	0.96 (0.64, 1.43)	0.831
	Decrease	epidemic concern	1.11 (0.88, 1.40)	0.367
		impact of psychology	1.32 (1.05, 1.65)	0.016
		impact of life	0.87 (0.68, 1.11)	0.265
		impact of work or study	1.07 (0.85, 1.34)	0.554
	Uncertain	epidemic concern	0.57 (0.44, 0.76)	<0.001
		impact of psychology	1.56 (1.16, 2.10)	0.003
		impact of life	1.16 (0.84, 1.61)	0.357
		impact of work or study	1.10 (0.81, 1.48)	0.556

a Baseline of sugary foods intake was regarded as a stratification factor

b Unwillingness to change sugary foods intake was regarded a reference group

c Subjective impacts were classified into dichotomous variables in the models (regarding “low,” “poor,” and “medium” as 1 and others as 2), adjusting for other characteristic factors (gender, age, BMI, education level, marital status, occupation, and main living place)

## DISCUSSION

The ongoing COVID-19 outbreak has become a global pandemic. Millions of people are at risk of infection of the rapidly spreading virus, which has already impacted local residents on different aspects (e.g. psychology [15], social and socio-economic [4, 5]) in both the affected and non-affected areas, posing an unknown health threat globally. Public health recommendations and governmental measures during the COVID-19 pandemic have enforced numerous restrictions on daily living including social distancing, isolation and home confinement. However, these measures may cause negative change of lifestyle behaviors, including less physical activity and unhealthy diet that place individuals at higher risk of chronic disease and leaving them more vulnerable to COVID-19 [12]. In this study, we aim to explore the relationship between subjective perception of impact due to COVID-19 and willingness to change dietary habits during the outbreak of the COVID-19 based on a cross-sectional study in China.

The average age of the subjects surveyed in this study was 27.9±7.8 years old. Those with middle or above concerns about COVID-19 reached 84.5%, implying that the respondents had a high overall focus on the epidemic. The epidemic clearly has affected the psychology (60.2%), life (66.3%), and work or study (66.8%) among the respondents, which suggests that the impact of the epidemic on the population far exceeds the pathogenic harm of the infectious disease itself. At present, psychosocial impact of COVID-19 and psychological interventions have aroused wide public concern [16]. It has been shown that the outbreak has significantly increased psychological problems, such as depression and anxiety [3], and health care workers [17], pregnant women [18], and the elderly [19] are more susceptible. Based on a cross-sectional survey among 7,236 self-selected volunteers, Yeen Huang et al. reported that young people who spent too much time focusing on the COVID-19 epidemic information every day were at a high risk of mental illness [3]. This indicated that epidemic concern might be an important factor that contributed to psychological impact. Thus, timely mental health care and living support are urgently needed for the general public.

It is worth noting that the prevalence of chronic diseases in patients with COVID-19 is higher than the estimated national prevalence [20]. Patients with chronic diseases are more likely to be infected and had higher mortality than healthy people [7]. It’s known that the main factor that promotes the occurrence of chronic disease is an unhealthy lifestyle [13]. In this study, we found that the COVID-19 pandemic changed respondents’ dietary habits. The results of the willingness to change dietary habits showed that the proportions of people reducing intake of salt, fried foods, sugary foods were 31.9%, 46.0%, and 41.0%, respectively. According to knowledge, attitude, and practice (KAP) of lifestyle is a continuous, interconnected, and long-term process [21], our results implied an early stage of positive improvement of adopting healthy diets after the pandemic period in China. This might attribute to the self-conscious adoption of healthy lifestyle practices in order to improve health status and prevent infection. A network investigation on KAP about COVID-19 among 4,016 residents in Anhui Province, China, reported that, compared to lifestyles of “no gathering and less going out”, “wearing masks when going out,” and “not going to crowded and closed places,” etc., the ratio of residents that could achieve the lifestyle of “light diets with balanced portion of vegetables and meat” was relatively low (65.6%) [22]. So, it is of great importance to enhance residents' awareness of healthy diets under new situation of the pandemic.

It is seen that psychological factors, including perceived severity, confusion about information reliability, were associated with responses of recommended and avoidance behaviors during the outbreak of COVID-19 [15]. However, we observed significant associations between psychological impact and the willingness to increase poor dietary habits. Regarding unchanged willingness as a reference group, respondents with higher psychological impact were more likely to increase their dietary intakes of salt, fried foods, sugary foods, after adjusting for gender, age, BMI, education level, marital status, occupation, and main living place (Tables 6–8). This implies that psychological impact might be the common factor for adopting poor dietary habits. People with increasing psychological pressures tend to adopt poor diets [23]. The negative changes in the majority of eating behaviors could be attributed to eating out of anxiety or boredom, or an increase in anxiety or mood driven eating [24]. Studies have found that most young people tend to consume more high-salt, high-energy fast foods under stress, and people with lower self-efficacy have higher intakes of fried foods, sugary drinks, and sweets [25]. Thus, health care and lifestyle support or interventions for young people should be given more consideration.

For those with low-salt diets before the epidemic, respondents with higher epidemic concern (OR 0.75, 95% CI 0.60–0.95) and higher impacts on work or study (OR 0.68, 95% CI 0.54–0.86) were less likely to reduce their salt intakes. For those who ate less fried foods (<1 day/week) before the epidemic, respondents with a higher impact on life were less likely to reduce eating fried foods (OR 0.79, 95% CI 0.66–0.93). Furthermore, for those ate less sugary foods (<1 day/week), the stratified analysis yielded similar results of the associations between subjective impacts and changing willingness to those without fried foods (Tables 4–6). This may be associated with the following reasons: 1) Epidemic concerns and perceptions of impacts on life, work were interrelated with psychological perceptions; for example, young people paying too much attention to the epidemic were at high risk for mental illness [3]. 2) During quarantine, more young people have tended to choose convenient and fast food or takeout food, increasing their frequency and quantity of snacks [26]. 3) Because of the bias of self-reported, actually some respondents might use to higher intake of salt, fried foods, sugary foods than they reported [27]. Thus, some of those recorded as having a light diet before the epidemic might be more susceptible to poor dietary habits during the outbreak of COVID-19. Further study is needed to understand the changing characteristics of dietary habits. Except as aforesaid, most respondents with higher epidemic concerns and impacts on psychology, life, work were more likely to reduce eating salt, fried foods, and sugary foods.

## CONCLUSION

Our study had several notable limitations. Firstly, the findings from this brief cross-sectional study are only suggestive (not confirmative) for causal associations between subjective impact and willingness change to dietary habits. Secondly, all indicators were based on an online survey of respondents’ self-reports and were thus subject to recall and report bias. Thirdly, we did not use standardized scales to assess subjective impact indexes (e.g. subjective perception of epidemic concern and impacts on psychology, life, work during the COVID-19 outbreak) and baseline and change willingness of dietary habits (e.g. the status of salt intake, the frequency of eating fried foods, and sugary foods) due to a limited survey time. This may have limited the comparison of our findings with previous studies. Lastly, we might have missed some groups lacking interest in these kinds of online surveys or lacking access to a social media.

Although this convenience sample is not representative of the public at large, the anonymity, confidentiality, and sample size of the data may partially overcome the factors mentioned above. In summary, our findings suggest that subjective impacts were substantially high in China during the outbreak of COVID-19, and poor dietary habits, such as high-fat, high-sugar, and high-salt diets were found among some of the respondents. After the epidemic, a positive improvement to a proper diet was observed, whereas the opposite tendency was also found some respondents with higher impact on psychology. Psychological impact might be an important factor for adopting poor dietary habits, interrelated with epidemic concern and impact on life, work. Moreover, those who had a light diet but higher subjective impact might be more susceptible to poor dietary habits. Thus, health care and lifestyle interventions for different subgroups should be made for local residents in China.

## MATERIALS AND METHODS

### Participants

The cross-sectional study was conducted online for all netizens in China in March of 2020. The participants were recruited by the snowball sampling which is a nonprobability method with advantages of convenient operation, high efficiency [14]. The inclusion criteria include (1) they volunteered to participate in this questionnaire survey, and (2) they independently completed the questionnaire with no logical errors. A total of 28,877 respondents in China completed their questionnaires in the study. Among them, 1,966 dropped out of the study, and 26,911 validly completed their questionnaires.

### Data collection

The questionnaire was published in the WeChat public account of Bao'an District Hospital for Chronic Diseases Prevention and Cure. All questionnaires were completed online by logging onto a web address or scanning a QR code. At the beginning of the survey, we used a unified guidance language to introduce the study purposes and also ensured data confidentiality to the respondents. When there was any omission or logical error, the system prompted the respondent until the questionnaire was completed and submitted. The online questionnaires were anonymous so that the respondents could not be affected by any other factors in expressing their opinions, which helped to obtain more authentic and reliable data than traditional paper questionnaires. Of the 26,911 respondents, 4,452 invalid questionnaires (with logical errors) were excluded, and finally 22,459 were effective; the effective rate was 83.5%.

### Questionnaire

A structured questionnaire (Tables 2, 3) with close-ended questions was developed after literature search [15] and consulting experts, and included the following: (1) General characteristics: gender, age, height, weight, education level, marital status, occupation, main living place during the outbreak of COVID-19, etc.; (2) Subjective impacts: self-rated degree (none, low, medium, high, higher) of subjective impact indexes, including subjective perception of epidemic concern and impact on psychology, life, work during the outbreak of COVID-19; (3) Baseline of dietary habits before the COVID-19 outbreak: salt intake (high, medium, and low), frequency of fried foods intake (times per week), and sugary foods intake (times per week) which consisted of sugar, sugary beverages, desserts, cookies, candies, fruit products, dairy desserts. (4) Willingness to change dietary habits: self-reported change willingness (unchange, increase, decrease, uncertain) of food intake, including salt, fried foods and sugary foods during the COVID-19 outbreak or later.

### Statistical methods

The original data of the questionnaires filled in online were downloaded directly from the website, and all the data were imported into SPSS18.0 software for statistical analysis after the invalid questionnaires were removed. Measurement data (age and BMI) followed a normal distribution, were described using the metric means and standard deviation, and were analyzed by the Student's t-test to compare the means between different groups. Categorical data were described as the proportion and analyzed with the chi-square test. We treated salt intake (low=1, medium=2, and high=3) and frequency (<1 day/week=1, 2–3 days/week=2, 4–6 days/week=3, every day=4) of eating fried foods, sugary foods as ordinal variables. Gender was treated as a dichotomous variable, while education level, marital status, occupation, main living place, and willingness to change dietary habits was regarded as nominal variables. Subjective impacts were classified into dichotomous variables (“none,” “low,” and “medium” were defined as 1, while “high” and “higher” were defined as 2), while the baseline of the diet was regarded as a stratification factor and unchange willingness as a reference group in the multinomial logistic regression models, in order to investigate the effects of subjective impacts on willingness to change dietary habits, after adjusting for other characteristic factors (gender, age, BMI, education level, marital status, occupation, and main living place). For all analyses, a p-value <0.05 was regarded as statistically significant.

### Ethics approval

This study was approved by the ethics committee of Guangdong Medical University.
